# Fusing Object Information and Inertial Data for Activity Recognition †

**DOI:** 10.3390/s19194119

**Published:** 2019-09-23

**Authors:** Alexander Diete, Heiner Stuckenschmidt

**Affiliations:** Data and Web Science Group, University of Mannheim, 68159 Mannheim, Germany; heiner@informatik.uni-mannheim.de

**Keywords:** activity recognition, machine learning, multi-modality

## Abstract

In the field of pervasive computing, wearable devices have been widely used for recognizing human activities. One important area in this research is the recognition of activities of daily living where especially inertial sensors and interaction sensors (like RFID tags with scanners) are popular choices as data sources. Using interaction sensors, however, has one drawback: they may not differentiate between proper interaction and simple touching of an object. A positive signal from an interaction sensor is not necessarily caused by a performed activity e.g., when an object is only touched but no interaction occurred afterwards. There are, however, many scenarios like medicine intake that rely heavily on correctly recognized activities. In our work, we aim to address this limitation and present a multimodal egocentric-based activity recognition approach. Our solution relies on object detection that recognizes activity-critical objects in a frame. As it is infeasible to always expect a high quality camera view, we enrich the vision features with inertial sensor data that monitors the users’ arm movement. This way we try to overcome the drawbacks of each respective sensor. We present our results of combining inertial and video features to recognize human activities on different types of scenarios where we achieve an $F_{1}$-measure of up to 79.6%.

## 1. Introduction

Human Activity Recognition is an active field of research in pervasive computing [1,2,3,4]. One popular task in this field is the recognition of so-called activities of daily living [5]. As the cost for care increases [6,7,8], many fields like health care and nursing could benefit from computer-aided solutions that support care givers. Often, these problems are solved using smart home environments where activities are inferred from ubiquitous sensors in the living area, giving care givers information more easily. However, these approaches can be very costly, as they often have to adapted to each environment separately and require a fairly large infrastructure. Additionally, the task of recognizing individual activities gets harder if a shared living space is used as sensor events can be attributed to multiple people. With the increasing market of smart-devices many sensors are becoming widely available and cheap. We propose the usage of such off-the-shelf smart-devices to recognize aforementioned activities, where we rely on inertial sensors and an ego-centric camera for our prediction.

Several studies already investigate activity recognition, be it low-level [2,9,10] or high-level activities [3,11,12]. Usually, the former comprises actions, such as *standing*, where the latter refers to context-enriched actions such as *preparing food*. To recognize the later, researchers often rely on head mounted cameras built into devices such as smart-glasses. Their results show that object-based activity recognition is one of the most promising vision-based approaches [13]. However, the object recognition itself is error-prone and crucial in respect of the recognition quality [11] and is therefore crucial to the whole pipeline. Smart-phones and smart-watches that are equipped with accelerometer, gyroscope, and magnetometer are another popular choice. In contrast, however, inertial-based high-level activity recognition approaches usually perform less accurately but are a reliable option for low-level activities. This also includes the tracking of the user’s arm [10] which we need for our approach. Therefore, many researchers started to adapt the approach of fusing multiple sensors to get a better overall result. Approaches to fuse the inertial and vision sensors have already been made by other researchers [14]. However, most of the work focuses on the fusion of sensor streams that belong to the same on-body position [3,15] and rarely looks at different body positions. Complex activities have often been detected using smart environments with sensors attached to objects and location to recognize interactions. Such approaches can give exact results regarding an interaction with an object but are expensive to deploy in real-world scenarios, given the high amount of variation in home environments. Especially with goods such as food, placing such sensors may not be feasible on a bigger scale. This can be seen in practice in the currently tested retail shops created by Amazon [16] where good results are achieved but the number of sensors needed is very high. In addition, an interaction with an object that was registered via a sensor may not translate to a properly performed activity (for example if a pill box was touched by a user but no medicine was consumed).

In this paper, we present our work on a multimodal ego-centric activity recognition approach that relies on smart-watches and smart-glasses to recognize high-level activities, such as the activities of daily living. For that purpose, we combine inertial and video information and try to take advantage of each of their strengths. In particular, we consider the inertial data of our smart-watch to classify the movement pattern of the forearm. The video data provides object information from the smart-glasses. We aim to investigate to what extend vision information can improve the recognition of activities that are hard to recognize purely through motion sensing. This is especially the case when motions are short or very similar (e.g., eating vs. taking medicine). We present the results of our multimodal activity recognition approach based on manually annotated video data. In addition, we test our approach on a public dataset that contains data from similar sensors but set in another scenario. Specifically we look at the CMU-MMAC dataset [17] that contains recordings of people cooking different recipes. Our contributions in this publications are:

1. We collected a new dataset with two subjects performing a set of activities in two different environments with a focus on activities that are hard to distinguish as they involve similar motions (e.g., eating and drinking) and are often interleaved. Each subject performed the activities in different human body positions and at different speeds. Currently there are few datasets that cover these scenarios; thus, other researchers in the field can test their approaches on this dataset.
2. We present a new method and a baseline comparison for multimodal activity recognition, using deep learning models for object detection and evaluating this method on our presented dataset, achieving an $F_{1}$-measure of 79.6%. We also apply our method to the CMU-MMAC [17] dataset and can show that we outperform previous work on the same dataset. Additionally we tested our method with a greater subset of the CMU-MMAC dataset, as a recent publication offers more annotations [18].

This work is an extension of a previous publication [19]. In this extension, we included experiments with more data, a deeper analysis and a comparison of our work against another system.

## 2. Related Work

There are several methods and publications from the domains of image and video processing that target sub-problems of our research question. Similarly, using inertial data for activity recognition has also been researched in depth. The approaches in both of these fields have shown to perform well in their respective applications. In the following, we summarize methods that can be used to support multimodal activity recognition. Namely, we look at separate methods for vision and inertial data first and then at work for combining both of them.

### 2.1. Image Object Detection

In recent years, there have been advances in deep and neural network-based object detection. One prominent example is the TensorFlow Object Detection API (https://github.com/tensorflow/models/tree/master/research/object_detection), that integrates many popular architectures in one easy to use API. The API offers deep learning-based approaches for object detection that rely on pre-trained models which were initially evaluated on the Microsoft COCO object detection challenge [20,21]. Given an image, the TensorFlow model generates bounding boxes for potential objects and annotates them with object classes. Each annotation is associated with a confidence value, allowing users to work in depth with the data. Thus, we decided to use this framework for our methods. Many different neural network architectures are offered and have their separate advantages. One typical trade-off is between performance and run-time. A currently well performing network is NASNet with a Faster-RCNN [22] yields an mAP score of 43.1%. In our case, we rely on a ResNet FPN model as described in [23], as the reported performance of 35% mAP is still among the best offered. However, it offers the advantage of a run-time that is significantly lower than the state of the art network (1833ms vs. 76ms). Using these object information, we can work towards recognizing activities.

### 2.2. Activity Recognition Based on Objects

Using object information for activity recognition, especially when looking at complex activities, such as cooking for example, was explored by many researchers [17,24,25]. For this purpose, the occurrence of objects and possibly also the interaction with said objects are used to recognize an activity. Wu et al. [26] already showed good results by detecting change in objects position, using an RFID sensor as a way to validate the interaction. In this case, the camera was stationary, pointing towards the location of action, thus making the detection of change a feasible approach. Similarly, Lei et al. [27] build their system on a RGB-D camera system, detecting activities in a kitchen environment. Here, focus was put on the recognition of actions and objects, using tracking methods, and object detection. Adding a camera to a wrist-worn sensor is another approach for detecting activities and was analyzed by Lei et al. [28]. A wrist-worn camera has the added benefit of having interactions with objects always in frame. Also, the hand movement is synchronized with the camera movement, making reasoning about egomotion vs. outside movement easier. Recently, Kumar et al. [25] used off the shelf object detection network and transfer learning to find correlations between predicted object labels and ground truth data of activities. This approach is very promising, as it explores the transfer of deep learning models in vision to model for activity recognition. One problem image-based recognition models face in practice is a limited field of view of the camera. When an activity occurs that is not fully captured within the field of view, the information is lost to a system. Systems that use stationary cameras may not suffer too much from this issue but involve an initial setup of a smart environment and are less flexible in their usage. Therefore, we additionally look at inertial data which also has been used by many researchers to detect and recognize human activities.

### 2.3. Activity Recognition Based on Inertial Data

One main reason for the increased focus on inertial data for activity recognition is the rise in popularity of smart-devices which often have a series of sensors (including inertial sensors) built into them. In this context, inertial data typically refers to acceleration, gyration and magnetic field data. With these sensors in mind, a lot of research has been conducted on cyclical activities, such as walking, running, or resting where especially the position of the sensor on the body (e.g., pocket for phones or wrist for smart-watches) has been examined in depth. Sliding windows, in combination with acceleration data, is a typical method to predict activities and was analyzed by many researchers before [29,30,31]. In particular, activities, such as walking, jogging, and climbing stairs, have been predicted successfully. Hereby, the position of the acceleration sensor is one important factor that was considered for the prediction [30]. Sensors are often placed on legs, arms, and the torso of subjects and then evaluated either separately or in combination. Features that are calculated from these windows are often from the time and frequency domain and may consist of measures, such as mean and variance, but also more computationally expensive and complicated features like energy [2]. Apart from cyclic activities, researchers also use inertial data to detect short activities or events. A common use case for short activities is the detection of accidents, such as falls [32,33,34]. As our scenario also involves many short activities, these methods are interesting to our problem setting. Falling, however, is an activity with a unique motion that is hard to mix up with other activities of everyday living. Therefore, we cannot fully use the methods presented there and have to adapt them to our needs. Finally for classification, classifiers that are commonly used are Decision Trees [29], Hidden Markov Models [31], and kNN [30]. However, recently, Ordóñez et al. [35] also employed Neural Networks for similar tasks. Here, it was shown that by adding new modalities (for example gyration data on top of acceleration data) to a network, features can be extracted automatically without the need for manual pre-processing. By using convolutional layers in their network architecture, every added modality was adapted properly, without the need for manual feature engineering. In our work, we rely on a sliding window approach, similar to [2]. However, in contrast to low-level activities, where the window size can be fairly long thus capturing abstract characteristics of an activity and better dealing with noise, we rely on short windows with an overlap between consecutive windows. This way we aim to capture the short nature of our activities within the windows while also allowing for an easier fusion later. We looked at the separate methods for activity recognition using video and inertial data and are now looking at methods for fusing them together.

### 2.4. Multimodal Activity Recognition

Previous work presents multiple methods to combine sensors and to create and analyze multimodal datasets [17,36,37]. Scenarios recorded in the datasets vary greatly and involve activities such as office work [37], sport activities [36], and cooking [17]. Using these datasets, researchers developed and evaluated different methods to recognize activities. Spriggs et al. [14] for instance, fused vision and inertial data to recognize cooking activities. One problem that is central in dealing with multimodal datasets is the fusion of sensors with different sampling rates. Inertial data is usually sampled at a higher rate than video data, especially when using off the shelf sensors. Spriggs et al. [14] solved this problem by downsampling the inertial data to the capture rate of the video, thus having a one to one mapping of frames to single inertial measurements. When dealing with windowed feature, some of these problems can be mitigated. By defining windows via start and end time rather than number of instances, one central timeline for data from different sources can be used. This allows for an easier merging of the different modalities. Once a valid temporal mapping is available, the problem of fusion methods can be addressed. Song et al. [37] published their ego-centric multimodal dataset which contains video and inertial data from smart-glasses. To recognize life-logging activities, they developed and presented a fusion method for inertial and video data. They combine the modalities with Fisher Kernels and could reach a high level of accuracy. Other methods of fusing multiple modalities often rely on Kalman Filter [38,39] where the results are often used in the fields of robotics. In these scenarios however, the camera and the inertial sensors are located at the same place. Thus, both sensors capture the same motion. Our scenario has the inertial measurement unit capture the movement of the arm, while the camera is located on the subjects head, thus such fusion techniques may not be easily applicable.

In this work, we rely on a windowing approach for both of our modalities. By aligning them and then using windows defined by a timespan, our data can be merged and we can evaluate late and early fusion approaches for our task.

## 3. Dataset

In this work, we look at two separate datasets to test and evaluate our developed methods. The first dataset was collected by us and deals with a subset of activities of daily living. It focuses on activities that are hard to distinguish just based on the inertial data, as they involve very similar motions. The second dataset we looked at is the CMU-MMAC dataset which contains a wider variety of activities with more test subjects. Namely, the dataset has recordings of people preparing different recipes in a test kitchen environment. In the next two subsections both our dataset and the CMU-MMAC dataset are described in regards to content, size, and target classes. We also describe a new set of annotations for the CMU-MMAC dataset that was published recently. For our own dataset we also go into detail about the recording process of the data.

### 3.1. ADL Dataset

For this dataset, we recorded two test subjects performing different activities in a typical home environment. All the recordings were done in an experimental setting and the test subjects consented to having the data recorded and published. Furthermore, we remove the audio track from all video recordings and cut away all video data that was not part of the scenario. Egocentric video was obtained from two angles: via smart-glasses and a chest mounted tablet. Additionally, we recorded the test subject from a third person view and used the video for the annotation of activities. The subjects were also equipped with smart-watches and smart-phones to capture the movement of their arms and thighs. Here, we recorded acceleration, gyration, and magnetic field data for all sensors simultaneously. This way, we only focus on the scenario and leave out any conversations or other interactions of the subjects within the test environment. The subjects performed common and interleaved activities which include *drinking* ($A_{1}$), *eating* ($A_{2}$), *taking medicine* ($A_{3}$), *preparing meal* ($A_{4}$), *taking snack* ($A_{5}$), and *wiping mouth* ($A_{6}$).

**Figure 1:**
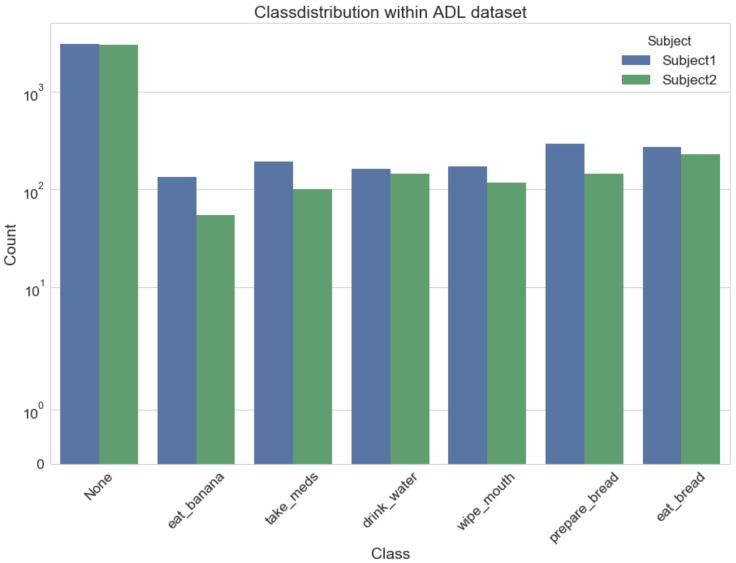
Distribution of classes per test subject using logarithmic scale as the majority of class labels belong to the none class. It can be seen that the majority class (excluding the none class) changes for each subject.

The procedure of the recording sessions was predefined as the whole dataset is not too big in size and variation would make proper classification unfeasible. Hence, the subjects executed two certain sequences ($A_{5}$, $A_{3}$, $A_{1}$, $A_{5}$, $A_{6}$ and $A_{4}$, $A_{2}$, $A_{3}$, $A_{1}$, $A_{2}$, $A_{6}$) where each sequence was performed two times. Once, it was done in a natural fashion and the other time with short interruptions between the individual activities. This way, we have scenarios where the activities are easy to separate and others where they are slightly overlapping. As these activities can be performed in several different postures, i.e., standing, sitting, and partly also lying, we recorded several sessions for each posture separately. To add more complexity, the home environment of the recordings switches between two different locations, adding different backgrounds to the video. Overall, we recorded six sessions per subject which results in 30 min of activities. Figure 1 shows the distribution of classes among the subjects in the subset of sitting activities. The difference in distribution can be attributed to differences in performing an activity, where *subject1* for example was taking more time to butter their bread than *subject2*.

**Figure 2:**
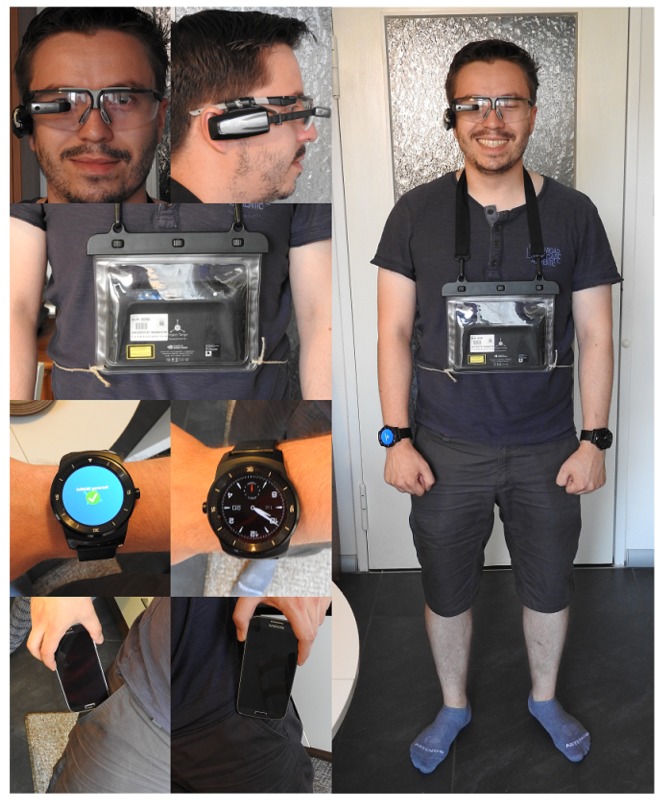
Sensor placement. The subject wears the wearable devices on the head, chest, forearm, and thigh (top down).

**Figure 3:**
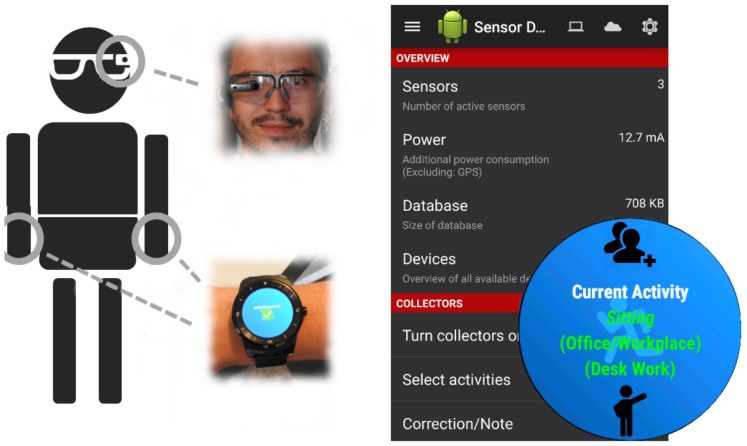
Sensor data collector application. The application is able to record a big set of sensors in Android devices including inertial data, temperature, and audio for example.

The required data was collected using different smart-devices (“Vuzix M100” (Glasses), “LG G Watch R” (Watch), “Tango” (Tablet), “Samsung Galaxy S4” (Phone)) (see Figure 2) which were attached to the head ($P_{1}$), the left ($P_{2}$) and right ($P_{3}$) wrist, the chest ($P_{4}$), and also to the left ($P_{5}$) and right ($P_{6}$) thigh. Video and inertial data was recorded with a resolution of 1920 × 1080 (25fps) and 50 Hz, respectively. In this context, the parameters were chosen with reference to related studies [1,29]. Data was collected via an app that we developed (see Figure 3). Each device was running an instance of the app and stores the data it receives in a local sqlite database. Other sensors, such as temperature, audio level and others, can also be captured via the application presuming the device has said sensors built into it. The binary and the source code (https://sensor.informatik.uni-mannheim.de/#collector) for the recording application are publicly available.

**Figure 4:**
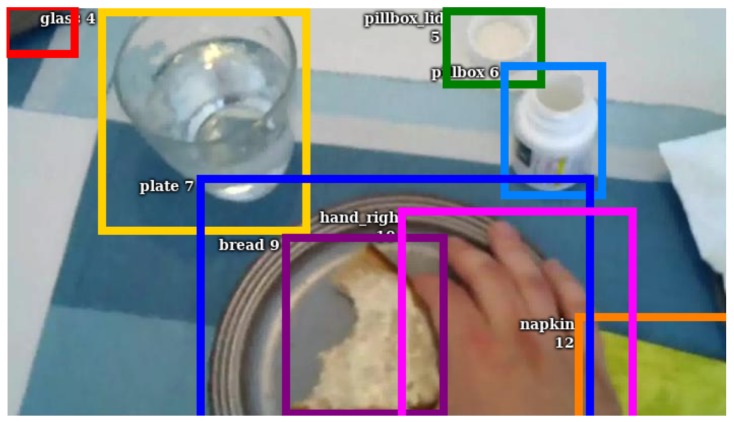
Example bounding boxes. It depicts a usual frame that was captured by our smart-glasses. We draw the bounding box for each object, even if it was only partly visible. The boxes were tagged concerning the visibility state of the object.

After the recording, we manually annotated the collected data on an activity level defined via start and stop time and the performed activity. Annotations are based on the third-person recording of the data as this view fully captures the motion of the test subject. Egocentric vision can often leave out the proper start and end of an activity as the field of view of the camera does not allow fully capturing all the movements. For labeling the activities, we used the *Behavioral Observation Research Interactive Software* [40]. On an object level, we drew the required bounding boxes around the visible objects within the ego-centric video of the smart-glasses. In this context, we marked 14 objects including *bread*, *napkin*, *glass*, *knife*, *pillbox*, and both hands. Figure 4 shows an example of the bounding boxes and also highlights that most objects are usually blurred or partly out of frame. Labeling of bounding boxes was done with *vatic* [41].

Data that was recorded on the same device (i.e., the smart-watch paired to the smart-phone) uses the same clock (namely the internal clock of the android phone) thus does not to be aligned. However, to further work with the data, we had to align all sources of data to be able to work within one consistent time-space. To be able to align the data easily, the test subjects started the recording with a period of no movement. This way, we could pinpoint the start of the movement for each sensor and therefore could calculate the time difference among them. We annotated the start of the motion with the *boris* annotation software for both the ego-centric and third-person video. Simultaneously, we mark the same point in time in the plot of the acceleration data and store the resulting timestamp. Using the alignment points, the activity labels can be mapped to any of the collected sensor data. This means, we can assign each frame of a video a timestamp that is consistent with the timestamps of the acceleration data.

Our labeled dataset is publicly available including a detailed description and images of each subject and the environment (https://sensor.informatik.uni-mannheim.de/#dataset_firstvision). In this work, we rely only on the smart-glasses and smart-watches. With such a setup, we try to maximize the recognition performance but still use a fairly small number of sensors.

### 3.2. CMU-MMAC: Quality of Life Dataset

The Quality of Life dataset [17] was created by the Carnegie Mellon University and contains a large set of test subjects, cooking a total of five different recipes. Modalities that were recorded include first person overhead video, inertial measurement units that record acceleration, gyration, and magnetic field data on different body positions, audio from five different microphones, and in some cases even motion capturing data. With the recording of so many different sensors, synchronization becomes an issue. The authors have used two different methods to address this issue. First, the recordings of the sensors we consider in our experiment (video and inertial data) was done centrally on one laptop that the subject is carrying with them. This way, single frames and readings of the inertial data are synchronized on one device as they use the time of laptop. For the rest of the data, synchronization among the devices was achieved by synchronizing the clocks on all computers with the NTP protocol.

For our main analysis, we focus on a subset of recipes, the brownie recipe, as labels for these recordings are provided by the original authors. We use this dataset to analyze different challenges and questions that cannot be addressed in our own dataset. One question is the behavior of our model when trained on a larger dataset. As we only have two test subjects in our recordings, we want to use the CMU dataset to test our method on a bigger dataset. The subset of the Quality of Life dataset contains thirteen different test subjects compared to our two different test subjects. This yields more variation as more subjects are performing the activities and in total also more data to train and evaluate our model. Another challenge is the complexity of the labels which is already obvious due to the more complex scenario of cooking. Annotations are given in the form of *verb-object1-preposition-object2*. Here, the brownie recipe consists of 17 different verbs, 34 different objects and 6 different prepositions. Overall we counted 43 different labels in the subset we considered. With so many classes, and some of them only having a few instances, a learned model would overfit to these instances resulting in biased numbers. Thus, we decided to group the labels in some form to create a meaningful model. To achieve this, we only look at the verb part of the activity as our target class. In the scenario of the CMU dataset, combinations of activities are nearly endless and our proposed method targets a closed set of activities (e.g., taking medicine in the ADL scenario). While vision information is needed to determine the objects used in the activity, only the verb part really benefits from both inertial and vision data. Our assumption is that activities with the same verb share common movement patterns but only in combination with the vision information we can distinguish some classes. This reduces the number of classes to 14 and also allows us to compare our method to previous methods as in [42] who also used only the verb part of the activities.

In total we looked at 13 different subjects, only considering the overhead camera frames and the acceleration data on both arms in our analysis. Data was aligned and trimmed with the provided synchronization files, and afterwards cut to the length of the sequence of activities.

### 3.3. CMU-MMAC: New Annotations

Recently, a new set of annotations for the CMU-MMAC dataset was released that vastly increased the number of labeled scenarios. In [18], the authors showed their approach for annotating the data while also offering semantic annotations that can be used in other experiments, e.g., when using reasoning. Overall they added annotations for three recipes and for all subjects, with the exception of cases where the video files were broken and could not be used. Annotations are mostly based on the first person view, thus making it easy to use with our previous approach.

**Figure 5:**
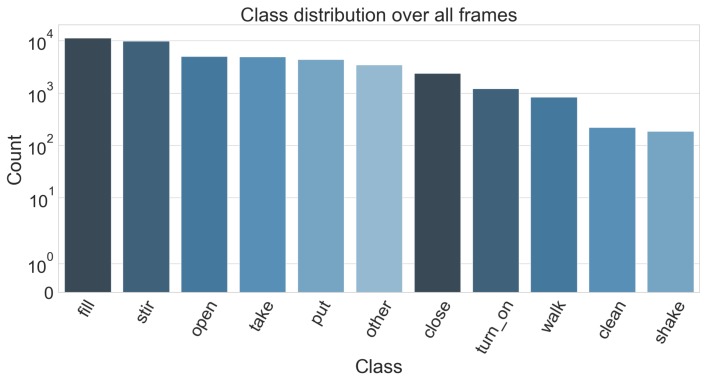
Distribution of the classes we consider from the CMU-MMAC dataset using the annotations from [18]. The class label is derived from the verb part of the original label.

To make learning activities feasible, we only considered one recipe: baking brownies. This way, we alleviate an issue with the annotations and our problem description. Labels are given in a similar fashion as whey are on the official CMU MMAC dataset website. Namely, the use the form *verb-object1-object2-...-object_n* to properly specify the activities, where the number of objects can vary depending on the scenario. An example would be the class (*open drawer*) vs. (*fill oil oil_bottle pan*). However, this once again yields a huge number of different labels (in the subset of brownie recipe, there is total of 165 different annotations) which in turn makes learning each one of them unfeasible especially since 59 of these labels have less than 10 instances in the dataset. Instead, we are also only considering the verb part of these annotations (see Figure 5). When we look at all recipes however, there are a lot of cases where a verb is used with objects that are unique for each recipe. This in turn makes the group of activities with the same verb very heterogeneous, thus making it difficult to learn the specifics of an activity. Therefore, to make the learning feasible and also compare the results to the original annotations, we only look at the complete set recordings for the brownie recipe.

## 4. Methods

### 4.1. Acceleration Data

**Figure 6:**
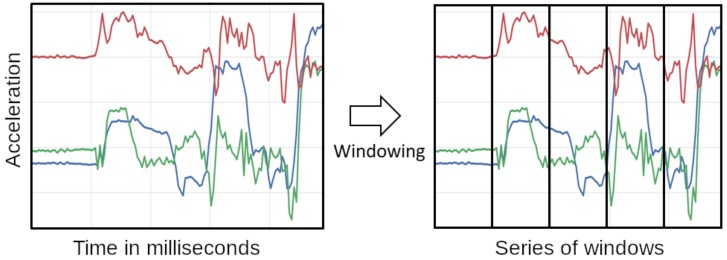
Windowing of inertial data. Windows have a length of 1s and an overlap of 50% or 75%.

To keep the number of used sensors minimal, we only consider acceleration data from the smart-watches. Activities we aim to recognize are mostly performed with the hands, allowing us to only consider said wrist-worn sensors. Other inertial data that may be interesting for activity recognition in our scenario is the data collected by the smart-glasses. One example for using smart-glasses inertial data may be to give a better understanding about when a subject is moving their head to take a sip of a cup. Initially, we planed to only consider data form the dominant hand of the test subjects, but as activities were often performed with a mix of both hands, we decided to use both. For our features, we use a sliding window approach. Figure 6 visualizes the windowing of inertial data. To generate the windows we use a framework we developed that is publicly available (https://sensor.informatik.uni-mannheim.de/). Here, the framework first takes all data for one modality and calculates temporal windows based on a set of parameters it receives. Thus, we transform the time series into a set of discrete windows which allows us to analyze them separately. Temporal windows have the advantage that they can contain different amounts of data points per window in contrast to typical windowing approaches which are defined by the quantity of points they contain instead of a timespan. Defining the window size via a timespan makes the approach more robust in potential real-world settings as sensors may drop single readings which would result in a shifting of the windows. After the windows are defined, data points are added to the windows and each window calculates a set of features (described in Table 1). Some of the features are in the time domain others in the frequency domain. Generally, the prediction power of features can vary as we showed in previous work [43], but in this scenario we kept all features and let the learning algorithm decide which ones to use. For the parameters of the windowing we set the length of the windows to 1000 ms and the overlap to 50% or 75%, depending on the scenario. We base our settings for the window size on previous works in the field [2] as well as adapting it to the scenario. Longer window sizes than 1000 ms would not be feasible, as the activities we consider are very short and longer windows would contain multiple activities. Shorter window lengths on the other hand have difficulties to capture enough specifics about the movement to properly distinguish the activity. The window overlap allows us to look at the dataset with a finer resolution which, given the short nature of some of the activities, can be very useful. For motions such as raising an arm towards a glass or picking up items, inertial data could be sufficient. However, to properly detect the different activities, we also have to consider the visual information as acceleration information may not differentiate between picking up medicine vs. picking up food.

### 4.2. Video

Video features in our model are based on object information within the frames. To recognize the objects, we use a pre-trained object detection neural network and transform its results into feature vectors. As described in Section 2, we use bounding boxes of a ResNet FPN network. Masks of the objects were also considered initially. However, the added benefits of more details are outweighed by the significantly longer run times for detection and the comparison with our ground truth, which is present in bounding box format, not being ideal. When looking at the activities from the first person view, we could see that a main component of the activity is the interaction of the test subject with different objects. We assume that interactions with different objects are a good indicator for an activity and we were also able to see that in an initial experiment. Here, labeling the interaction with a video annotation tool, and using these interactions as a feature vector, we could show a very high performance of the model (close to 100% accuracy). It thus can be seen that recognizing interaction of a person with their environment should be a main goal of our approach. Our estimation of an interaction works by looking at the overlap of bounding boxes from a detected hand and any other type of object. Therefore, we first pre-filter the frames and only consider the frames that contain a positive detection for a hand (which is labeled as a *person* within the target-classes of our neural network). In these frames, we then calculate the overlap of each detected object’s bounding box with the hand’s bounding box. This results in a vector of the length of objects classes that can be detected by the neural network minus the people class (as an overlap of the hand with itself does not add any information). The rest of the frames are assigned to a vector of the same dimension filled with negative one as a value for each interaction. For each frame, we thus get a feature vector that describes which objects are present in a frame and how much they overlap with the the detected hand.

To further work with the generated image features (and especially combine them with the inertial data) we apply another windowing approach to the image features. Here we consider a window of frames where we calculate the average overlap of each object with the hand within the window where the window size is ten and the stride is five. As within the video a hand often hovers over different objects when performing an activity, overlaps are often calculated even though no interaction with any objects occurred.

**Figure 7:**
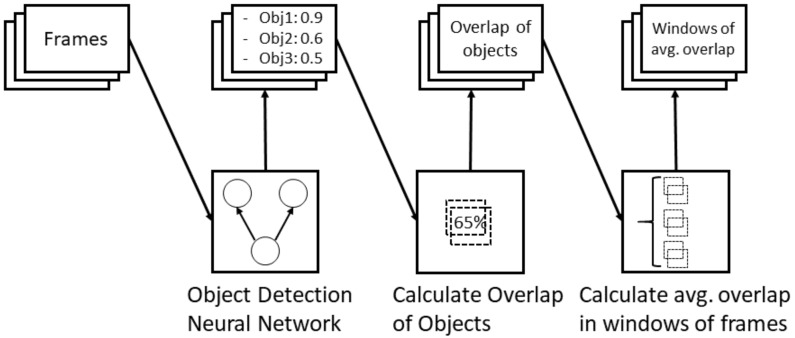
Pipeline for the image feature generation.

We assume that interactions with objects yield a longer span of time where the detected hand overlaps with the object thus the mean overlap within a window is greater than an overlap of the hand when passing an object within the frame. The whole process of extracting vision features is described in Figure 7.

To evaluate the approach further, we ran the experiments on learned image features as well as on object annotation ground truth data. This way we can analyze the reliability of the vision features without dealing with wrong or missing classifications from our object detection network. As we do not have object annotations for the CMU-MMAC dataset, this step could only be done our own dataset.

Given the two modalities, we now try to estimate the activity the subjects perform. For that purpose, we define a method to combine both features to be used in one machine learning model. Before we combine the data, we first have to align both modalities which is described in Section 3. Since the data may start at different times, we consider the biggest temporal overlap of the data. Here, we consider the latest starting point and the earliest ending point among all modalities for each scenario. Points of data that are earlier or later than the respective starting and end points are not considered for the experiment and are discarded. The resulting data will later be used for training and testing. From the trimmed data we calculate our features as described before. As our windows have temporal information, we can map the windows to each other and have one consistent dataset. Consider as an example a video window $w_{vid}$ with start end end points $t_{s}$ and $t_{e}$. By considering the starting points of the inertial windows, we can find the starting point $t_{s}^{\prime }$ which minimizes the temporal distance $t_{s}-t_{s}^{\prime }$ where $t_{s}^{\prime }\geq t_{s}$.

**Figure 8:**
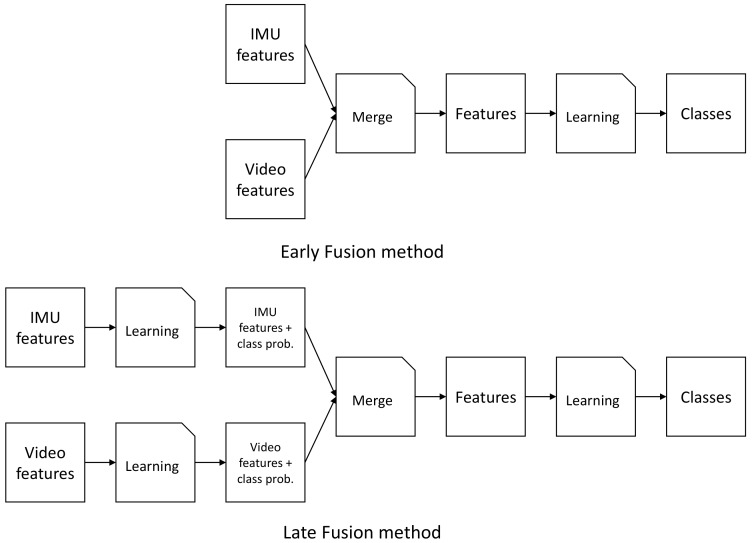
Pipeline for the fusion of the modalities. The top pipeline shows our early fusion method, the bottom one our late fusion approach.

The first approach we test is early fusion where we concatenate the feature vectors of all three modalities and learn one model. This way, all information is immediately present in the model and classifiers can chose what features to use. Figure 8 shows a simple flow diagram of the fusion approach (seen at the top). For simplicity, the inertial data was shown as one flow, though we use two sensors there. As fusion of the modalities happens before any model is learned, this pipeline can be seen like a typical machine learning problem where one dataset is used to train and test a model.

### 4.3. Combining Both Modalities

Anther approach is late fusion learning, seen at the bottom of Figure 8. Here, we first concatenate both inertial windows and lean a model for this features subset. Simultaneously, we learn a separate model for the image windows. For both modalities we return the class probabilities and append them to their respective feature vectors. Finally, we concatenate both feature vectors with the added probabilities to one big feature vector. Using these combined features, we learn a final model to predict the activities. This approach separates the modalities first, thus giving each model the chance to learn specifics for each modality. Furthermore, we can leverage different machine learning algorithms for each modality. As the features for each modality represent different aspects of the activity, using separate algorithms could be beneficial for the overall results as different algorithms may be better suited to cover different modalities.

For more insights, we evaluated the combination as well as both sensors separately in our experiments section. To gain more insights about each modality, we report the performance of each sensors separately in addition to the final performance. This way, we can also see if the different modalities work best with different learning algorithms. The next section describes the experiments in greater detail and presents the results.

## 5. Experiments

### 5.1. ADL Dataset

For the experiments, we consider each subject separately and test our model with a cross-validation. A cross-subject setting could be used with a bigger dataset but since this dataset includes two subjects it is not feasible to learn a model this way. To test for stability in the smaller datasets we run each cross validation 100 times with different folds and check for similar results. As we want to have a deeper insight into the influence of each modality in combination with different classifiers, we test different combinations of classifiers for the multimodal settings. Configuration parameters include the classifier that is used for the late fusion learning, which modalities are used, and whether ground truth or the neural network bounding boxes are used. For classification, we use Random Forest and Logistic Regression algorithms. We also tested other classifiers, such as SVM, but the results were most promising with the above mentioned algorithms. When we consider all modalities, the classifiers used for the separate sensors are Random Forest for acceleration data and Logistic Regression for vision data. This way we keep the single modalities fixed and only change the fusion learning algorithm, reporting it’s performance at the end. We also tested early fusion, but this yielded an overall performance loss for the classification in our cross-validation evaluation.

Using a sliding window approach with overlap poses a problem: two consecutive windows may end up in the training and in the testing set respectively. Since windows are overlapping, theoretically these overlapping parts of the data are part of windows in both training and testing. To avoid this, we sampled our data depending on which modalities we evaluate, making sure that no data is present in training and testing simultaneously. In both the vision and combined approach, the windows are based on the vision windows. As it has an overlap of 50%, we consider every other window and, in the case of the combined approach, the respective IMU window. When considering only acceleration data, the overlap of windows is 75%, thus we consider every fourth frame in the experiments. In this specific case, the amount of data available can be fairly small for some of the very short activities, resulting in folds with very few instances of some classes. Therefore we used a five-fold validation in these scenarios instead of a 10-fold cross-validation. The results are reported as an average of both test subjects.

Table 2 shows that the best configuration uses all modalities and Logistic Regression as the fusion learning algorithm, yielding a $F_{1}$-measure of 79.3% (leaving out the ground truth vision scenarios). As expected, the results for using only vision features are far higher when assuming perfect vision. The gap in performance can most likely be attributed to the current state of object detection algorithms. Especially with our scenarios including different environments and a camera sensor of lower quality, pre-trained object detection still lack behind. With a bigger dataset, a custom model could be trained that may improve on the vision results. Considering the results of the inertial data classification, a great difference in performance among the learning algorithms is visible. This is in line with the analysis of related work that showed that Random Forest classification works well with inertial windows [2]. The combination of the sensors however is helping the results overall. Especially given the fact that these results are achieved with a pre-trained object detection model. Overall, the results of the classification tend to prefer a high precision at the cost of recall which is beneficial in our scenario as a sequence of correctly classified activities with some windows not assigned at all can still be used to reconstruct the correct timeline. In the next step, we take a closer look at the separate classes and their performance using the best configuration from the previous experiment.

Table 3 shows the results for all classes, broken down for each class separately. On a first glance it can be seen that the performance varies among the different activities. Great performance can be achieved for the bread preparation class, with an $F_{1}$-measure of 90.9%. One possible explanation for the good performance is the uniqueness of the features for both modalities we considered. In the case of inertial data, the motion of buttering a piece of bread is distinctively different from the other activities which all involve some sort of grabbing or lifting motion. For the video data, this scenario also offers unique views, as the test subjects were looking down on their plate and focusing on it and the bread. Most of the other classes are performed with an overlook of the table, thus resulting in a similar scenery. Additionally, the class, in combination with eating bread, was performed the longest by the subjects yielding more instances for training.

Eating a piece of banana and wiping the mouth after the full sequence were the worst performing activities, yielding $F_{1}$-measures of 64.3% and 68.8% respectively. There are separate reasons for both classes. In the case of eating a piece of banana, the shortness of the activity is the main problem. Test subjects were eating just one piece of fruit which was readily available on the table. Thus the activity is very short, only offering a few unique aspects to be learned. Wiping the mouth has the issue of hard to detect objects. The napkin was often only partially visible, parts of it hidden underneath a plate. This makes it difficult for the vision features to detect the object.

### 5.2. CMU-MMAC Dataset

For the experiment of the CMU-MMAC dataset with the original annotations, we evaluated the whole dataset among all subjects to see how well a model can be applied among a set of subjects instead of learning per single subject. Here we also ran the experiment 100 time and calculate the average precision, recall and the resulting average $F_{1}$-measure.

Given the harder task of the CMU-MMAC dataset, we achieve a lower $F_{1}$-score of 58.4% (see Table 4). This is not surprising given the setting of the dataset, where a larger number of subjects perform a greater set of activities which both adds more variation to the data. What contributes to this fact, is the larger number of subjects performing a greater set of activities, both of which adds more variation to the data. The bad performance using the vision features is also striking, with the performance going down to 32.1%. One explanation for this score is the reduction of the annotations to just the verb part. Annotations for the dataset were provided in the form of *verb-object1-preposition-object2*. As this results in a very huge set of labels with small amounts of instances per label, we reduced the annotations to just the verb. Thus, activities, such as *open-brownie_box* and *open-cupboard_top_left*, are assigned the same label, even though they are performed on very different objects and in different situations. Vision features in this context are relying on the objects visible in frame and thus have issues to properly differentiate the different activities. What is also striking is the fact that on this dataset the vision features performed better with a Random Forest instead of the Logistic Regression as it was the case in the ADL dataset. When looking at the acceleration data though, the results are fairly good. This is in line with results in [44] where it was shown that hierarchical clustering of the activities tends to favor activities with the same verb. Therefore, acceleration data is able to represent similar activities in a similar fashion. However, in the context of this dataset, Logistic Regression does not seem to be able to properly learn a model for the inertial data. We could already see that Logistic Regression performs worse on our dataset when applied to acceleration data. This effect is even stronger in the CMU-MMAC dataset, most likely because of the bigger set of labels that have to be recognized. Random Forest behaves similarly in both cases and yields good results which is in line with previous research [2]. As object annotations for the videos are not present in the CMU-MMAC dataset, we cannot run experiments on perfect vision.

To look deeper into the classification results, we consider the IMU classification results on their own and show the performance for each class.

Table 5 shows our findings. Good performance can be seen in classes, such as pouring and stirring, with a $F_{1}$-score of 58% and 74% respectively, while generic classes, such as reading or closing, are not recognized very well. This seems to be in line with our assumption that the acceleration data is able to distinguish specific activities (i.e., stirring involves a motion that is very unusual compared to the others) and has problems distinguishing verbs that are very generic.

To compare our results we evaluate against a previous approach [42] that uses the same scenario for their dataset (i.e., the brownie recipe of the CMU-MMAC dataset) and also the same approach for reducing the labels. They use a novel classification approach on SIFT features from the video-frames of the dataset. To fit the evaluation of the work, we modify our training to use the first eight of the test subjects for training and the last four for testing. In this scenario, we also used re-sampling of the data to simulate an even class distribution. We report the results of for the $F_{1}$-measure for each class.

**Figure 9:**
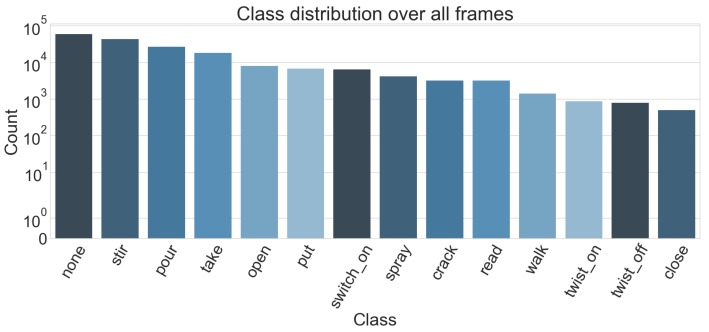
Distribution of the classes we consider from the CMU-MMAC dataset. The class label is derived from the verb part of the original label.

Table 6 shows that with the exception of the pour and the none class, our approach outperforms previous results. Overall, this evaluation setting shows a performance drop, as we consider a fixed split that only allows for a small trainings set. This way we are also encountering the difficult problem of cross-subject learning, which we did not consider in the previous experiments. What can be seen though is that some classes, such as stirring, putting and taking, can be learned across subjects given enough training-data. Evidently, these are also among the classes that occurred the most in the dataset (see Section 3, Figure 9).

We can see that the combination of inertial and video data yields a better result than each sensor on its own. Depending on the activity that should be recognized, modalities perform differently as they are relying on the variation within the data. Inertial data for example, may not be as expressive when the activities that are performed are very similar in motion. Thus, it makes sense to consider the combination of both modalities to predict high level activities.

### 5.3. CMU MMAC: New Annotations

Next, we consider the new annotations provided by [18] to learn on an even bigger set of activities for the CMU-MMAC dataset. As done with the original annotations, we tested early and late fusion approaches in this setting. For our experiments, we considered the annotations for the Brownie scenario with 28 different test subjects. With the increased dataset however, it was also possible to run a gridsearch on the dataset to properly tune the classifier. Here we used a fixed split for training and test data with a split of 80% training and 20% testing data. Then we ran a gridsearch with a 5-fold cross-validation on the training data for each classifier, finally evaluating on the test dataset. For the random forest we tuned these parameters:

- Number of estimators
- Maximal depth of trees
- Min samples per leaf and per split
- The number of features to consider when splitting (all, or $\sqrt{n\_features}$)

For the logistic regression we considered:

- Number of iterations
- Optimizer type (newton, simple)
- Distance C

Results with the new dataset improved (see Table 7), with the new best model improving the $F_{1}$-measure by 8%. After running all experiments, we could see that the performance for logistic regression was worse than the random forest. These results differs from the previous experiments. It suggests that the logistic regression cannot fully abstract on a bigger dataset and thus the random forest is the overall better choice. Fully comparing the results is difficult however, as the annotations for the dataset are similar to the original but not the same. For the next step, we again look at the performance of the single classes to see if a similar pattern can be seen.

Table 8 shows the results of our run on the greater subset of CMU dataset for each activity separately. Overall the results are very promising and show an improvement to the previous experiments. This makes sense as the dataset size is greatly increased. Some trends that could be seen in the previous experiments are also present in the results of this experiment. Stirring, a class with a very unique motion and long sequence of data, can be recognized fairly well. Classes like walking though, are still hard to classify as they do not have enough inertial cues. Considering a sensor that is attached to the legs may yield better results but would increase the overall number of sensors which is why we left it out. A direct comparison to the original data is difficult though, as the annotations are not done by the same annotators and also use different classes. It can be seen though, that even the classes that were very difficult to classify with the original annotations (e.g., walking and closing) have improved with the bigger dataset. Overall, using a fused approach with a multimodal setting seems to be promising to classify human activities.

## 6. Discussion

The results of the experiments show that combining vision and inertial data is a promising approach for classifying human activities. It is helpful, especially in those cases where either of the modalities is not capable of capturing specific aspects of an activity. An example could be the consumption of a snack compared to the intake of medicine where an inertial sensor may have problems to distinguish the activity, as it relies to some extend on the objects used. However, the approach can still be extended. Estimating interaction with an object is one important aspect. Using the overlap of a hand with an object can yield good results but especially in frames with many objects, a lot of overlap can exist. In these cases motion tracking information of objects could help as it could be used to additionally detect the movement of an object. However, motion tracking is especially difficult in a scenario, where egomotion of the camera is present which is the case in the datasets we consider. Furthermore, as there is no depth information present in the data, an overlap cannot fully represent the interaction.

Another aspect to consider in this work is the issue of privacy. Systems that recognize activities always bear the challenge of privacy concerns, especially when video cameras are used in the process. When a video camera is recording a user or from a users perspective for a long period of time, it may capture activities that are deemed sensitive. We believe that smart-devices can help to mitigate the privacy concerns that arise when using cameras. For one, processing and calculation of the data can be done offline within the home environment where such a system is installed. Additionally, when on-the-fly classification becomes feasible, video data may not even be stored but just processed as a stream, in the end only using object information. In that context, if the set of objects that can be detected is kept to a minimum, the amount of sensitive information processed can be reduced greatly. This way, potentially no data is leaked to the outside which may help to mitigate possible concerns. Another aspect could be the use of the smart-device itself to recognize the context of a user. The camera could for example be turned off when a user is in a certain room or at a certain time where their privacy concerns are very strong (e.g., in context of personal hygiene).

We also considered using other sensors for the recognition, like depth or infrared cameras that may seem less intrusive at a first glance. The obvious downside of these devices is their relative low availability in smart-devices, making it difficult to easily use them with current technology. Depth cameras for instance, have become more common in smart-devices in recent years but still are not as prevalent as standard cameras. Additionally, the amount of sensitive information collected by these types of cameras is comparable to that of a standard camera and in some cases even higher, making the privacy concerns an even harder problem. Infrared cameras for instance can relay much more information about a person that is recorded just by the temperature data it collects. On top of that, there are also practical issues in our scenario. Depth cameras for example, are bound to a minimum and maximum distance they are able to capture. With a person wearing such a camera for an egomotion recording, many interactions close to the user may not be captured by the camera. Overall the usage of cameras can be challenging in a live system, but we believe that, considering the added information gain of the modalities and using a proper and privacy-aware implementation, such challenges may be overcome.

## 7. Conclusions and Future Work

In this paper, we presented a new multi-modal dataset that includes activities of daily living. It poses the challenge of similar activities, namely food and water consumption and medicine intake. All activities in the dataset were performed by two subjects at two different locations. The collected data includes acceleration, gyration and magnetic field data from six different body positions and videos from three different angles, two of which are ego-centric. Based on this dataset, we present a method for recognizing activities, using window features with fused video and acceleration data. Here, we use time and frequency domain features for the acceleration data and object information encoding hand interaction for the vision data. For the recognition of objects in a frame we use a pre-trained neural network where we use the overlap of the subjects hand and objects in a frame as a feature. After learning a model for each modality separately, we fuse them together and learn an overall model using Random Forest and Logistic Regression classifiers. This way we were able to achieve an $F_{1}$ measure of 79.6% on our presented dataset and 58.4% on the CMU Multi-Modal Activity dataset (66.4% for the bigger subset). We also show that we beat a state of the art activity approach for the CMU-MMAC dataset. Both scenarios (ADL and cooking) pose different challenges for our approach. For our dataset the similarity of the activities is challenging when considering acceleration data, as the difference of the actions is mostly rooted in the interaction with different objects. The CMU-MMAC dataset contains a wider variety of activities by a greater number of subjects, thus including more variation in the data. We can show that our approach is promising for the recognition of activities in a multimodal setting, including the usage of off-the-shelf sensors build into smart-devices. Especially when using the new bigger set of annotations, we could see that results improve.

In future work some aspects of the method could be varied. For the features, parts like the object detection network could be adapted. If we are able to get bigger sets of object annotations also for the CMU dataset, transfer-learning a model may be a feasible approach to get better object information. We could also re-evaluate the selection of modalities. So far, we focused on a relative small subset of modalities to analyze as we want to use as few devices as possible. Still, it may be interesting to evaluate different and greater sets of modalities for our goal, keeping in mind not to over-fit the approach. Gyration and magnetic field data are obvious candidates, as they are recorded alongside the same sensor already. Fusion techniques could also be changed, where for example different lengths of windows are used for each modality and between this bigger set of overlapping windows boosting or voting mechanism learn the best fusion strategy.

## Figures and Tables

**Table 1:** Set of features from acceleration data. Features are in the time and frequency domain.

Time Domain	Frequency Domain
Mean, Median, Standard Deviation, Variance, Inter Quantil Range, MAD, Kurtosis, Correlation Coefficient, Gravity, Orientation, Entropy (Time)	Energy, Entropy (Frequency), MeanDC

**Table 2:** Different configurations for our learning method. Values are reported as an average over all classes and for both subjects. RF = Random Forest, LR = Logistic Regression, ALL = both modalities were used, VIS = only vision features, IMU = only acceleration features, GT = ground truth vision, LEARN = vision features that were detected by our neural network.

Config	Precision	Recall	$F_{1}$-Measure
RF_IMU	0.673	0.556	0.609
LR_IMU	0.516	0.392	0.446
RF_VIS_GT	0.872	0.622	0.726
LR_VIS_GT	0.855	0.590	0.698
RF_VIS_LEARN	0.506	0.367	0.425
LR_VIS_LEARN	0.721	0.337	0.460
RF_ALL_GT	0.843	0.754	0.796
LR_ALL_GT	0.897	0.753	0.819
RF_ALL_LEARN	0.816	0.709	0.758
LR_ALL_LEARN	0.880	0.722	0.793

**Table 3:** An closer look at the results for our best configuration for each activity separately. Both vision and acceleration features were used in combination with Logistic Regression.

Class	Precision	Recall	$F_{1}$-Measure
none	0.928	0.986	0.956
drink_water	0.886	0.62	0.729
eat_banana	0.868	0.511	0.643
eat_bread	0.867	0.749	0.804
prepare_bread	0.891	0.929	0.909
take_meds	0.894	0.676	0.769
wipe_mouth	0.837	0.585	0.688

**Table 4:** Results for CMU-MMAC dataset. Here we used the same method as above to evaluate our method. As we do not have bounding-box ground truth data, we can only learn on the output of our neural network.

Config	Precision	Recall	$F_{1}$-Measure
RF_ALL	0.748	0.436	0.551
LR_ALL	0.738	0.482	0.584
RF_IMU	0.727	0.440	0.440
LR_IMU	0.230	0.115	0.153
RF_VIS	0.400	0.269	0.321
LR_VIS	0.395	0.395	0.295

**Table 5:** A closer look at our best performing configuration for the classes in the CMU-MMAC dataset. The model was learned in a 10-fold cross-validation among all subjects.

Class	Precision	Recall	$F_{1}$-Measure
close	0.516	0.062	0.111
crack	0.757	0.389	0.514
none	0.514	0.783	0.724
open	0.690	0.481	0.567
pour	0.601	0.613	0.607
put	0.752	0.460	0.571
read	0.834	0.551	0.664
spray	0.890	0.726	0.800
stir	0.744	0.811	0.776
switch_on	0.859	0.630	0.727
take	0.708	0.648	0.677
twist_off	0.824	0.188	0.306
twist_on	0.793	0.196	0.314
walk	0.695	0.215	0.328

**Table 6:** Comparison against state-of-the-art approach. Values marked with a (*) are directly taken from  [42]. Here the model is learned on 8 subject and tested on the remaining 4.

Class	Baseline (*)	SSVM (*)	PR-SSVM (*)	Our Approach
close	0.0	0.006	0.01	0.045
crack	0.065	0.035	0.053	0.124
none	0.075	0.195	0.251	0.198
open	0.098	0.124	0.152	0.181
pour	0.140	0.266	0.276	0.126
put	0.087	0.079	0.121	0.247
read	0.0	0.008	0.037	0.039
spray	0.016	0.013	0.016	0.074
stir	0.352	0.148	0.294	0.587
switch_on	0.038	0.043	0.042	0.098
take	0.075	0.195	0.139	0.234
twist_off	0.0	0.024	0.036	0.055
twist_on	0.0	0.02	0.025	0.047
walk	0.0	0.0	0.083	0.094

**Table 7:** Overall performance of different classifiers using early and late fusion. Late fusion dropped in this scenario.

Config	Precision	Recall	$F_{1}$-Measure
LR_EARLY	0.430	0.326	0.337
LR_LATE	0.378	0.323	0.329
RF_EARLY	0.831	0.604	**0.664**
RF_LATE	0.572	0.626	0.574

**Table 8:** Detail evaluation for classes in bigger CMU subset with best configuration which is random forest with early fusion.

Class	Precision	Recall	$F_{1}$-Measure
clean	0.947	0.409	0.571
close	0.764	0.479	0.589
fill	0.748	0.967	0.844
open	0.720	0.696	0.707
other	0.866	0.615	0.719
put	0.665	0.537	0.595
shake	1.000	0.270	0.426
stir	0.904	0.977	0.939
take	0.620	0.595	0.607
turn_on	0.976	0.840	0.903
walk	0.935	0.256	0.402
